# Inverse Association between High Blood 25-Hydroxyvitamin D Levels and Diabetic Retinopathy in a Representative Korean Population

**DOI:** 10.1371/journal.pone.0115199

**Published:** 2014-12-08

**Authors:** Donghyun Jee, Kyung do Han, Eun Chul Kim

**Affiliations:** 1 Department of Ophthalmology and Visual Science, College of Medicine, The Catholic University of Korea, Seoul, Korea; 2 Department of Epidemiology and Biostatistics, Harvard School of Public Health, Boston, MA, United States of America; 3 Department of Biostatistics, Department of Preventive medicine, The College of Medicine, Catholic University of Korea, Seoul, Korea; Massachusetts Eye & Ear Infirmary, Harvard Medical School, United States of America

## Abstract

**Purpose:**

To investigate the association between 25-hydroxyvitamin D and diabetic retinopathy (DR).

**Methods:**

A population-based cross-sectional study using a nation-wide, systemically stratified, multistage, clustered sampling method included a total of 18,363 subjects aged ≥40 years who participated in the Korean National Health and Nutrition Examination Survey during 2008–2012. All participants participated in standardized interviews, blood 25-hydroxyvitamin D level evaluations, and comprehensive ophthalmic examinations. Seven standard retinal fundus photographs were obtained from both eyes after pupil dilatation. DR was graded according to the modified Airlie House classification system.

**Results:**

The blood 25-hydroxyvitamin D levels were 19.2 ng/mL in men and 17.9 ng/mL in women. After adjusting for potential confounders, including age, sex, diabetes duration, hemoglobin A1c levels, and hypertension, the odds ratios (OR) for any DR and proliferative DR among men decreased significantly in the highest blood 25-hydroxyvitamin D level quintile relative to the lowest quintile (OR, 0.37; 95% confidence interval [CI], 0.18–0.76; *P* for trend  = 0.004 and OR, 0.15; 95% CI, 0.03–0.83; *P* for trend  = 0.043).

**Conclusions:**

This study provides the first epidemiologic findings of the inverse relationships of blood 25-hydroxyvitamin D levels with any DR and proliferative DR only in men. Considering anti-angiogenic and anti-fibrotic action of vitamin D, further studies including longitudinal and interventional analysis are warranted.

## Introduction

Diabetic retinopathy (DR), which is among the most common diabetes complications, is a leading cause of blindness among working-aged adults worldwide [1]. Major risk factors for DR include a longer diabetes duration, poor glycemic control, and hypertension, which have been strongly and consistently associated with DR across populations [2]. We previously reported that these 3 major risk factors could be applied to a representative Korean population, which is same data of the present study [3]. Other potential risk factors, including cardiovascular disease, lipid profiles, and obesity, have been inconsistently associated with DR across populations [4], [5]. Recently, an animal study found that the active metabolite of vitamin D, calcitriol, was a potent inhibitor of retinal neovascularization in an oxygen-induced ischemic retinopathy mouse model, suggesting that vitamin D may protect diabetic retinas [6]. Moreover, vitamin D has been implicated in the pathogenesis of type 2 diabetes mellitus [7]. Vitamin D deficiency has been shown to affect insulin synthesis and secretion in both human and animal studies [7].

Vitamin D, a circulating steroid hormone, has anti-angiogenic [6], [8], anti-inflammatory [9], [10], and anti-fibrotic properties [11], [12]. A number of studies have demonstrated the anti-inflammatory function of vitamin D in vitro and vivo [13]–[15]. Several human studies have shown inverse relationships between vitamin D levels and several chronic conditions associated with inflammation [16]–[19]. In the eye, vitamin D receptors are expressed extensively in the retina [20]. Therefore, vitamin D might prevent DR development and progression via its anti-inflammatory and anti-angiogenic properties. There is emerging evidence that DR is initiated and propagated by inflammation and angiogenesis [21], [22]. However, epidemiologic studies of the relationship between vitamin D and DR have been limited, although some clinic-based case-control studies have proposed a possible association between the 2 variables [23], [24]. To our knowledge, only 1 epidemiologic study has been performed to date. A study that incorporated the Third National Health and Nutrition Examination Survey (NHANES) data, which was completed 20 years ago, reported an association between DR severity and the prevalence of vitamin D deficiency. However, these findings were inconclusive with respect to an existing relationship between DR severity and the absolute serum vitamin D levels because the regression analysis did not show a significant relationship. We therefore investigated the relationship between 25-hydroxyvitamin D levels and DR in a large representative population of Korean adults.

## Methods

### Study population

**Figure 1:**
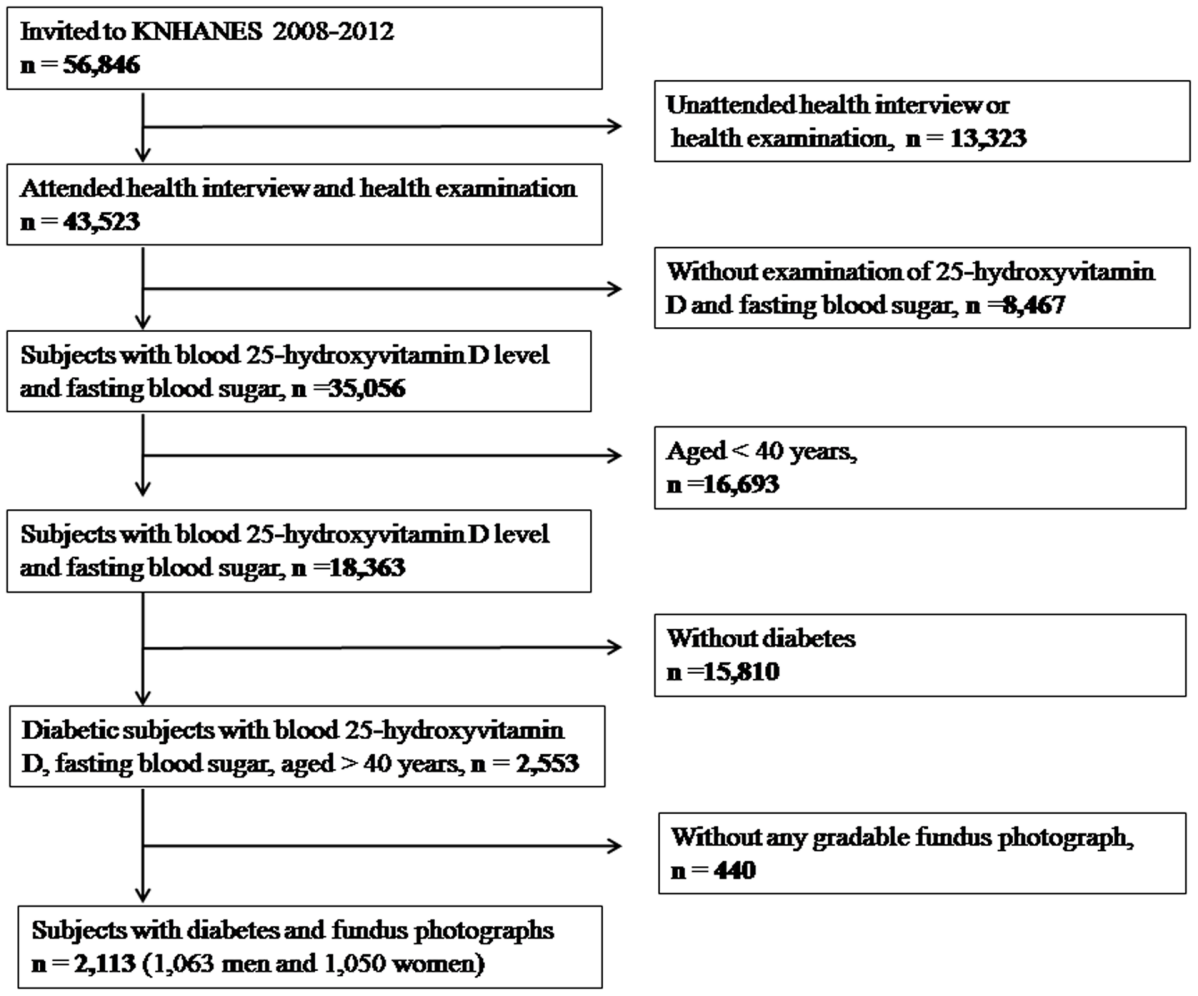
Flow diagram presenting the selection of study participants.

This study used data that were acquired for the Korean National Health and Nutrition Examination Survey (KNHANES). The KNHANES is a nation-wide, population-based, cross-sectional study conducted by the Korean Ministry of Health and Welfare and the Division of Chronic Disease Surveillance, Korean Center for Disease Control and Prevention. The KNHANES has adopted a rolling sampling design for a stratified, complex, multistage, probability cluster survey with proportional allocations based on the National Census Registry of the non-institutional civilian population of Korea. Details regarding the study design and methods have been provided elsewhere [25], [26]. Data from the fourth (2008–2009) and fifth (2010–2012) KNHANES were used to estimate the association between blood 25-hydroxyvitamin D levels and DR. For the current study, 35,056 individuals who underwent blood 25-hydroxyvitamin D level and fasting blood glucose level evaluations were selected. Of these, 16,693 subjects aged <40 years and 15,180 subjects who had not been diagnosed with diabetes were excluded. Of the 2,553 subjects with diabetes, 440 who did not undergo a fundus examination were excluded. Finally, 2,113 participants aged ≥40 years were included in the analysis (Fig. 1). The study design followed the tenets of the Declaration of Helsinki for biomedical research. The protocols for this study were approved by the Institutional Review Board of the Catholic University of Korea in Seoul, Korea. All participants signed and provided written informed consent.

### Data collection

A diabetes diagnosis was assigned to those with a self-reported history of physician-based diagnosis, those who were receiving drug treatment for diabetes, including insulin or oral hypoglycemic agents, or those with a fasting plasma glucose level >126 mg/dL [27]. Seven standard photographs were obtained for both eyes after pharmacological pupil dilatation according to the Early Treatment for Diabetic Retinopathy Study in participants with a history of diabetes, a random blood glucose level >200 mg/dL, or suspicious diabetic DR findings in non-mydriatic 45° digital fundus photographs (TRC-NW6S, Topcon, Tokyo, Japan) that were performed in all participants ≥40 years.

Details of the DR severity scoring method have been reported previously [28]. Briefly, DR was identified if any characteristic lesion as defined by the Early Treatment Diabetic Retinopathy Study severity scale was present; these included microaneurysm, hemorrhage, hard exudate, cotton wool spots, intraretinal microvascular abnormalities, venous beading, and new vessels. A DR severity score was assigned to each eye according to a modified version of the Airlie House Classification system [29]. The level of retinopathy was graded based on the worse eye. We defined 4 primary outcomes for this study on the basis of the severity scores in the worse eye. Any DR was defined as a level >14 and proliferative DR was defined as a level >60; clinically significant macular edema (CSME) was considered present when the macular edema was involved or within 500 µm of the foveal center or if focal photocoagulation scars were present in the macular edema. Vision-threatening DR (VTDR) was defined as the presence of severe non-proliferative DR, proliferative DR, or CSME [29]. Two different graders analyzed the fundus photograph images; a trained ophthalmologist acted as the senior grader and was consulted in cases of disagreement. These graders were masked to the clinical statuses of the subjects. The survey quality was verified by the Epidemiologic Survey Committee of the Korean Ophthalmologic Society. Training of the participating resident doctors was periodically performed by acting staff members of the National Epidemiologic Survey Committee of the Korean Ophthalmologic Society.

Blood samples were collected after 10–12 hours of fasting at the same day of taking the fundus photograph. Subsequently, the fasting glucose, hemoglobin A1c, total cholesterol, and triglyceride levels were measured using a Hitachi automatic analyzer 7600 (Hitachi, Ltd., Tokyo, Japan). 25-hydroxyvitamin D levels were measured using a radioimmunoassay kit (DiaSorin Inc., Stillwater, MN, USA) and a gamma counter (1470 WIZARD; PerkinElmer, Inc., Waltham, MA, USA). Details of the 25-hydroxyvitamin D analysis have been reported previously. [30], [31] All blood samples for analysis were properly processed, promptly refrigerated, and transported in cold storage to the Neodin Medical Institute (Seoul, Korea), a Korean Ministry of Health and Welfare-certified laboratory. The interassay coefficients of variation were 2.8–6.2% for the 2008–2009 samples and 1.9–6.1% for the 2010–2012 samples. KNHANES participates in the Vitamin D Standardization Program; therefore, the 25-hydroxyvitamin D measurements were standardized according to the recently developed National Institute of Standards and Technology-Ghent University reference procedure [32].

Blood pressure was measured with a sphygmomanometer with the patient in a seated position. After collecting 3 measurements at 5-minute intervals, we used the average of the second and third measurements for the analysis. The presence of hypertension was defined as a systolic blood pressure ≥140 mmHg, a diastolic blood pressure ≥90 mmHg, or the use of prescribed anti-hypertensive medication.

We collected demographic information during health interviews. We performed height and weight measurements while the participants wore light clothes without shoes. We calculated the body mass index as follows: weight (kg)/height (m)^2^. Age was stratified into 10-year intervals. The smoking status was self-reported, and the subjects were classified as current smokers, past-smokers, and never smokers. Alcohol use was self-reported, and the participants were grouped as ever drinkers or never drinkers. Data regarding the current sunlight exposure duration were obtained by selecting 1 of 2 options: <5 hours or >5 hours per day.

### Statistical analysis

The statistical analysis was performed using SPSS (ver. 18.0; SPSS, Inc., Chicago, IL, USA) to account for the complex sampling design. Strata, sampling units, and sampling weights were used to obtain unbiased point estimates and linearized standard errors. The participants' characteristics were described using means and standard errors for continuous variables and percentages and standard errors for categorical variables according to the presence of diabetes and DR. The analysis of variance or chi-square test was used to compare the demographic characteristics.

To evaluate the effect of the blood 25-hydroxyvitamin D levels on the DR prevalence, the blood 25-hydroxyvitamin D levels were categorized into quintiles [33]. Simple and multiple logistic regression analyses were used to examine the association between the blood 25-hydroxyvitamin D level and DR. After calculating the sex and age-adjusted odds ratios (OR; Model 1), we adjusted for age, sex, and other confounders, including the diabetes duration, hemoglobin A1c level, and hypertension, which have been established as independent risk factors in previous studies (Model 2) [2], [3]. For trend analysis, we evaluated change of ORs by increase of vitamin quintiles using quintiles as continuous variable. All logistic regression analysis variables were examined for multicollinearity, and only variables with a variance inflation factor <10 were used. *P* values were 2-tailed, and <0.05 was considered to indicate statistical significance.

## Results

Diabetes was diagnosed in 2,553 (13.9%) of the 18,363 eligible subjects aged ≥40 years who underwent blood 25-hydroxyvitamin D and fasting glucose level evaluations. Gradable seven standard photographs from the Early Treatment for Diabetic Retinopathy Study were obtained for 2,113 of the diabetic subjects (82.7%). Therefore, 2,113 subjects were included in the current study analysis. The demographic characteristics of the 18,363 enrolled subjects are summarized in Table 1 according to their diabetes and DR statuses. Subjects with DR were more likely to have a higher systolic blood pressure (*P* = 0.022), higher fasting glucose level (*P*<0.001), higher hemoglobin A1c level (*P*<0.001), and longer diabetes duration (*P*<0.001), compared to those without DR.

**Table 1:** Demographic and clinical characteristics, according to diabetes status and diabetic retinopathy (DR) status, as reported in the Korean National Health and Nutrition Examination Survey 2008–2012.

Characteristics	No Diabetes	Diabetes	*p*	Without DR	Any DR	*p*	Participants
	(n = 15,810)	(n = 2,553)		(n = 1,738)	(n = 375)		(n = 18,363)
**Male (%)**	47.8 (0.4)	55.2 (1.1)	<.001	54.9 (1.6)	54.4 (3.4)	.887	48.8 (0.3)
**Age (yrs)**	54.4 (0.1)	60.4 (0.3)	<.001	59.7 (0.3)	61.2 (0.6)	.059	57.4 (0.1)
**Body mass index (kg/m^2^)**	23.9 (0.0)	25.0 (0.1)	<.001	25.2 (0.1)	24.0 (0.1)	<.001	24.4 (0.1)
**Systolic blood pressure (mmHg)**	121.7 (0.2)	128.4 (0.4)	<.001	127.2 (0.5)	130.6 (1.3)	.022	125.0 (0.2)
**Diastolic blood pressure (mmHg)**	78.5 (0.1)	78.0 (0.2)	.059	78.1 (0.3)	75.9 (0.7)	.006	78.3 (0.1)
**Fasting glucose (mg/dL)**	94.7 (0.1)	142.6 (1.0)	<.001	141.4 (1.5)	157.8 (3.1)	<.001	118.6 (0.5)
**HbA1c (%)**	5.63 (0.0)	7.33 (0.0)	<.001	7.26 (0.0)	8.05 (0.1)	<.001	6.48 (0.0)
**Total cholesterol (mg/dL)**	194.3 (0.3)	188.0 (1.0)	<.001	187.1 (1.4)	185.8 (2.7)	.668	191.1 (0.5)
**Triglyceride (mg/dL)**	141.9 (1.2)	183.6 (4.6)	<.001	185.5 (7.2)	180.9 (8.2)	.683	162.8 (2.4)
**25-hydroxyvitamin D (ng/mL)**	18.7 (0.1)	18.8 (0.2)	.467	18.7 (0.2)	18.3 (0.4)	.380	18.8 (0.1)
**Diabetes duration (years)**	Non applicable	Non applicable		6.9 (0.2)	11.0 (0.4)	<.001	Non applicable
**Hypertension (%)**	36.1 (0.5)	60.4 (1.2)	<.001	60.6 (1.7)	55.8 (3.1)	.166	39.2 (0.5)
**Sun exposure (>5hrs/day, %)**	20.0 (07)	23.4 (1.3)	.001	21.1 (1.7)	19.3 (2.5)	.512	20.4 (0.7)
**Smoking status**			<.001			.935	
** Never (%)**	55.0 (0.5)	47.7 (1.2)		47.5 (1.7)	48.1 (3.4)		54.1 (0.4)
** Former(%)**	15.1 (0.4)	19.2 (1.0)		23.2 (1.4)	23.8 (3.0)		15.7 (0.4)
** Current (%)**	29.9 (0.5)	33.1 (1.1)		29.3 (1.5)	28.1 (2.9)		30.3 (0.4)
**Alcohol consumption (%)**	85.3 (0.4)	81.3 (0.9)	<.001	81.6 (1.2)	81.9 (2.3)	.902	84.8 (0.3)

Data are expressed as weighted means or weighted frequency (%) with standard errors. * p<0.05.

Gradable fundus photograph was obtained in 2,113 subjects (82.7%) of 2,553 subjects with diabetes.

The demographic and clinical characteristics according to the quintile blood 25-hydroxyvitamin D categories are presented in Table 2. As the blood 25-hydroxyvitamin D level increased, the participants were more likely to be male (*P* for trend <0.001), older (*P* for trend  = 0.016), smokers (*P* for trend  = 0.002), and to have a lower body mass index (*P* for trend  = 0.001), lower fasting glucose level (*P*<0.001), lower hemoglobin A1c level (*P*<0.001), and longer sun exposure duration (*P*<0.001).

**Table 2:** Demographic and clinical characteristics by quintile blood 25-Hydroxyvitamin D categories among representative Korean adults aged 40 years or older.

Characteristics	Quintile blood 25-Hydroxyvitamin D level (ng/mL)					
	13.0<(n = 425)	13.0–16.5 (n = 421)	16.5–19.6 (n = 423)	19.6–24.4 (n = 422)	24.4> (n = 422)	P for trend
**Male (%)**	41.4 (3.2)	50.9 (3.3)	57.4 (3.1)	64.1 (3.1)	60.7 (3.6)	<.001
**Age (yrs)**	69.8 (0.7)	59.0(0.6)	59.0 (0.5)	60.9 (0.6)	61.5 (0.7)	.016
**Body mass index (kg/m^2^)**	25.4 (0.2)	25.3 (0.2)	25.3 (0.3)	24.7 (0.2)	24.2 (0.1)	.001
**Systolic blood pressure (mmHg)**	129.3 (1.0)	127.2 (1.1)	126.3 (1.1)	128.2 (1.2)	128.5 (1.3)	.383
**Diastolic blood pressure (mmHg)**	78.3 (0.6)	77.6 (0.7)	77.6 (0.7)	77.3 (0.6)	77.7 (0.7)	.864
**Fasting glucose (mg/dL)**	147.6 (3.5)	144.7 (3.0)	152.7 (2.9)	139.0 (2.2)	137.3 (3.0)	<.001
**HbA1c (%)**	7.6 (0.1)	7.4 (0.0)	7.6 (0.1)	7.1 (0.0)	7.0 (0.0)	<.001
**Total cholesterol (mg/dL)**	188.2 (3.5)	190.9 (2.7)	187.0 (2.6)	185.2 (2.4)	182.6 (2.6)	.274
**Triglyceride (mg/dL)**	220.2 (23.9)	194.4 (10.4)	182.3 (9.2)	167.5 (7.3)	155.4 (7.2)	.004
**Diabetes duration (years)**	7.8 (0.5)	8.0 (0.5)	7.4 (0.4)	8.6 (0.5)	7.7 (0.4)	.417
**Hypertension (%)**	65.0 (3.4)	58.9 (3.1)	54.6 (3.1)	57.2 (3.3)	63.3 (3.5)	.143
**Sun exposure (>5 hrs/day, %)**	10.8 (2.3)	17.4 (2.5)	22.1 (3.2)	24.3 (3.0)	30.1 (3.6)	<.001
**Smoking status**						.002
** Never (%)**	57.4 (3.2)	47.9 (3.1)	50.4 (3.4)	38.3 (3.0)	42.8 (3.6)	
** Former (%)**	17.0 (2.6)	20.6 (2.6)	25.2 (2.8)	30.3 (3.0)	23.4 (2.9)	
** Current(%)**	25.5 (2.9)	31.5 (2.9)	24.4 (3.0)	31.3 (2.9)	33.8 (3.4)	
**Alcohol consumption (%)**	77.3 (2.6)	80.6 (2.5)	82.2 (2.4)	86.1 (2.1)	82.1 (2.3)	.141

* p<0.05.

The blood 25-hydroxyvitamin D levels in men and women according to categories such as age, DR, and sun exposure duration are presented in Table 3. The blood 25-hydroxyvitamin D levels were 19.2 ng/mL (95% confidence interval [CI], 18.7–19.8) in men and 17.9 ng/mL (95% CI, 17.2–18.6) in women (*P*<0.001). In women, the mean blood 25-hydroxyvitamin D levels were significantly higher in older subjects than in younger subjects (*P*<0.001); no corresponding significant difference was observed among the men (*P* = 0.371). In men, participants with diabetes had lower blood 25-hydroxyvitamin D levels (*P* = 0.006); no corresponding significant difference was observed in women (*P* = 0.321). For both sexes, the mean blood 25-hydroxyvitamin D levels were significantly higher in those receiving >5 hours of sunlight exposure per day than in those receiving <5 hours per day (*P*<0.001). There were no significant differences in the blood 25-hydroxyvitamin D levels between smokers and non-smokers of either sex.

**Table 3:** Gender difference of blood 25-Hydroxyvitamin D levels (ng/mL) of men and women according to age group and other variables among representative Korean adults aged 40 years or older.

Characteristics	Blood 25-Hydroxyvitamin D level (ng/mL)			
	Men (n = 1,063)	P	Women (n = 1,050)	P
**All subjects aged 40+ years**	19.2 (0.2, 18.7–19.8)		17.9 (0.3, 17.2–18.6)	
**Age groups**		.371		<.001
40–49 yrs	18.7 (0.7, 17.3–20.1)		15.7 (0.6, 14.4–17.0)	
50–59 yrs	19.0 (0.4, 18.0–19.9)		17.0 (0.5, 15.9–18.0)	
60–69 yrs	19.8 (0.4, 19.0–20.6)		19.2 (0.6, 18.0–20.5)	
70+	19.7 (0.5, 18.7–20.7)		18.6 (0.5, 17.5–19.8)	
**Diabetic retinopathy**		.006		.321
None	19.5 (0.3, 18.9–20.2)		17.7 (0.3, 17.0–18.5)	
Diabetic retinopathy	18.0 (0.4, 17.0–18.9)		18.6 (0.8, 17.1–20.2)	
**Hypertension**		.416		.833
Non-hypertension	19.0 (0.3, 18.2–19.8)		17.9 (0.4, 16.9–18.8)	
Hypertension	19.4 (0.4, 18.7–20.2)		18.0 (0.4, 17.1–18.9)	
**Sun exposure**		.001		.001
<5 hrs/day	18.7 (0.3, 18.1–19.3)		17.5 (0.3, 16.8–18.3)	
> 5 hrs/day	20.9 (0.6, 19.7–22.1)		19.8 (0.6, 18.6–21.1)	
**Smoking status**		.823		.105
Never	19.2 (0.6, 18.0–20.3)		18.0 (0.3, 17.3–18.7)	
Former	19.4 (0.4, 18.5–20.3)		15.8 (0.9, 13.9–17.7)	
Current	19.1 (0.4, 18.2–19.9)		17.9 (0.9, 16.1–19.7)	
**Alcohol consumption**		.768		.711
Never	19.0 (0.8, 17.3–20.6)		18.1 (0.5, 17.0–19.2)	
Ever	19.2 (0.3, 18.6–19.8)		17.8 (0.4, 17.0–18.6)	

Blood 25-hydroxyvitamin D levels were expressed as weighted estimates [%] (standard errors [%], 95% confidence intervals).

**Figure 2:**
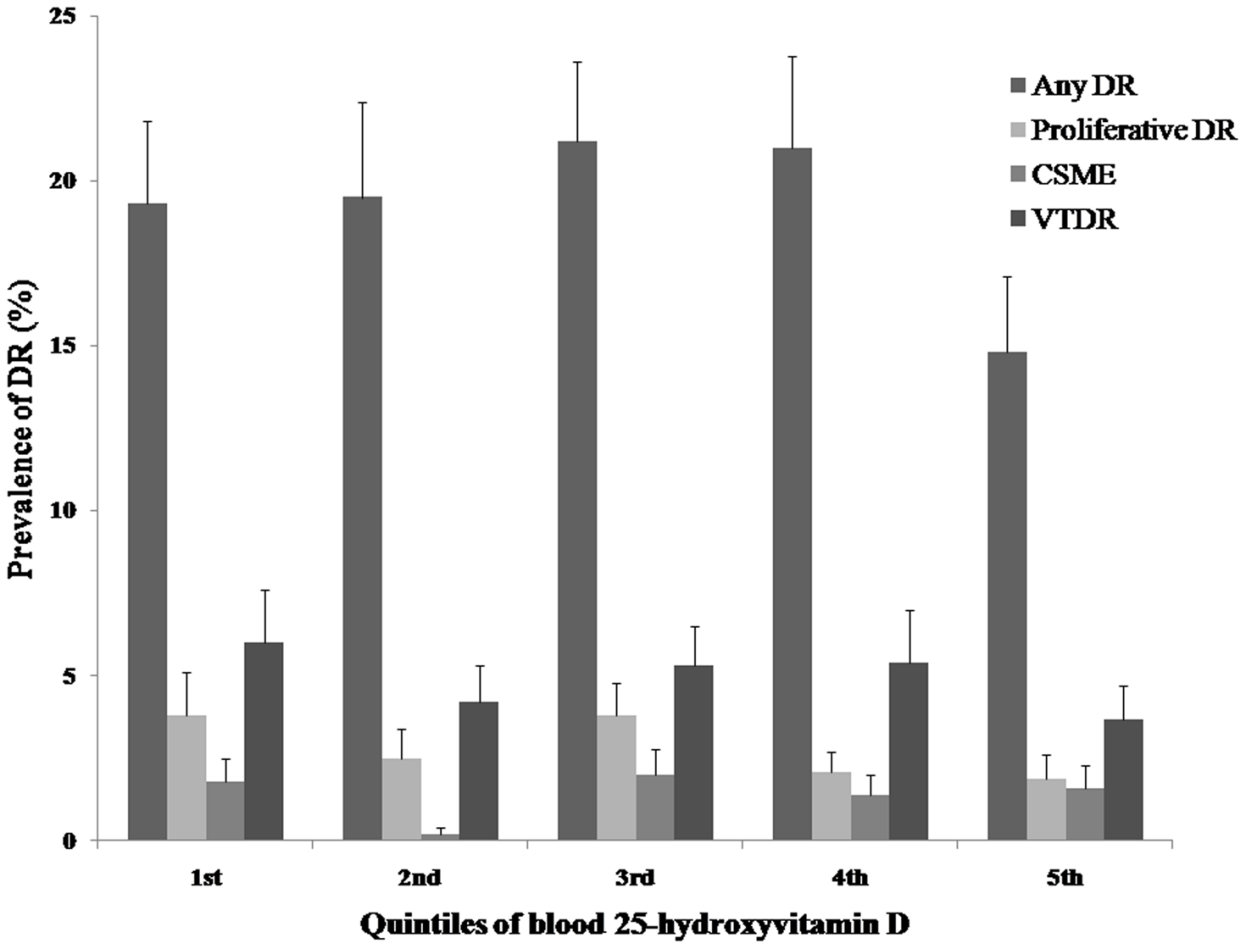
Prevalence of any diabetic retinopathy (DR), proliferative DR, clinical significant macular edema (CSME), and vision threatening DR (VTDR) according to the blood 25-hydroxyvitamin D level quintiles in a representative Korean population.

**Figure 3:**
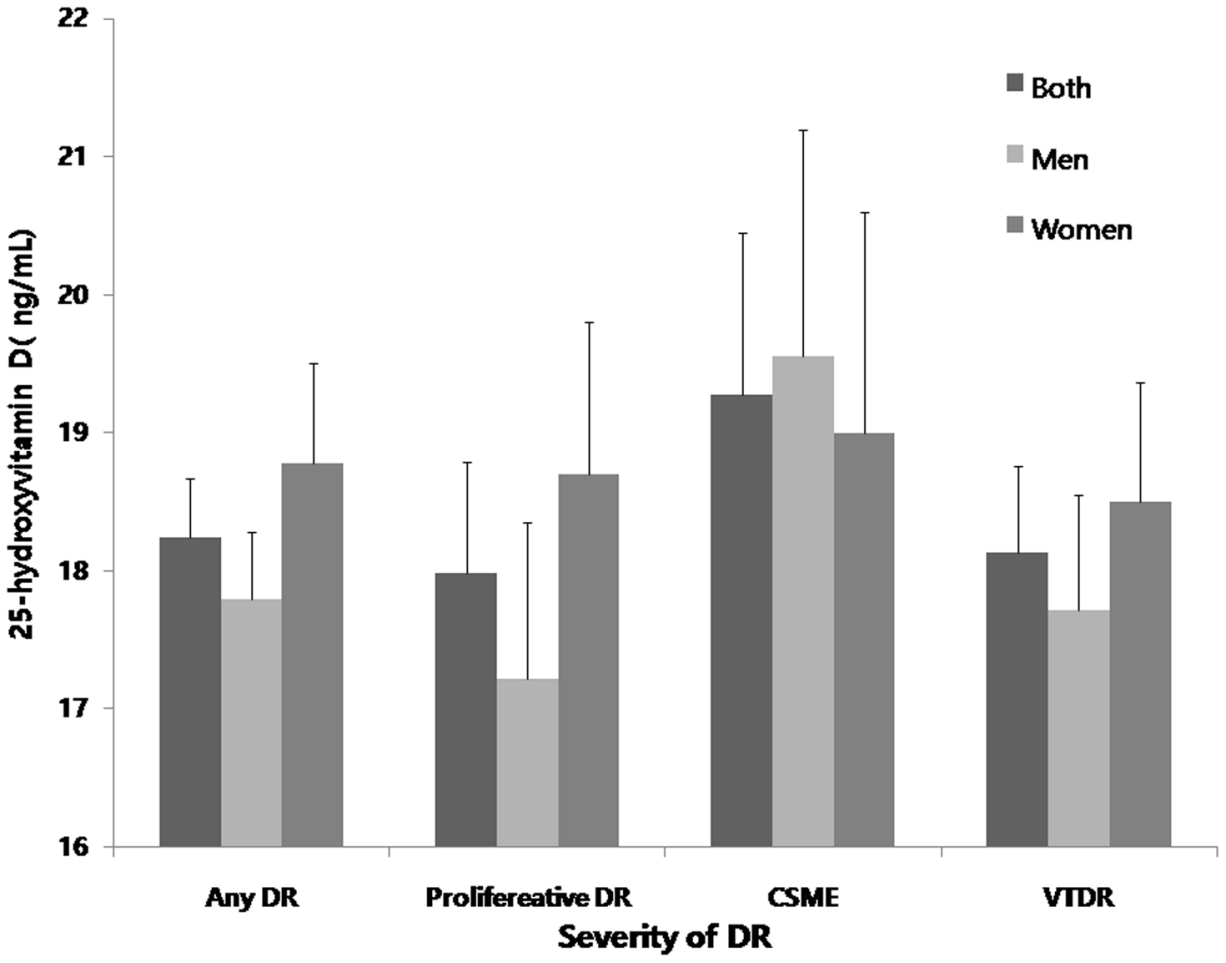
25-hydroxyvitamin D levels according to the severity of diabetic retinopathy (DR) including any DR, proliferative DR, clinical significant macular edema (CSME), and vision threatening DR (VTDR) in a representative Korean population.

The DR prevalence rates according to the blood 25-hydroxyvitamin D quintiles are shown in Fig. 2. As the blood 25-hydroxyvitamin D level increased, the prevalence of any DR decreased from 19.3% in the first quintile to 14.8% in the fifth quintile, although this difference was not statistically significant (*P* for trend  = 0.446). Additionally, the prevalence rates of proliferative DR and VTDR decreased from 3.8% to 1.9% and from 6.0% to 3.7%, respectively, in accordance with an increase in the blood 25-hydroxyvitamin D level, although this difference was not statistically significant (*P* for trend  = 0.404 and 0.697, respectively). Fig. 3 shows 25 hydroxyvitamin D levels according to the severity of DR and sex.

Table 4 lists the odds ratio (ORs) for the association of DR with the blood 25-hydroxyvitamin D levels. Overall, there were no significant associations between any DR, proliferative DR, CSME, and VTDR and the blood 25-hydroxyvitamin D quintiles after adjusting for potential covariates such as age, sex, diabetes duration, hemoglobin A1c levels, and hypertension. The adjusted OR for any DR, proliferative DR, CSME, and VTDR were 0.66 (95% CI, 0.38–1.13), 0.52 (95% CI, 0.16–1.74), 1.22 (95% CI, 0.40–3.72), and 0.64 (95% CI, 0.25–1.59), respectively, for participants in the highest blood 25-hydroxyvitamin D quintile relative to those in the lowest quintile.

**Table 4:** Prevalence and adjusted odds ratio of any diabetic retinopathy (DR), Proliferative DR (PDR), Clinically significant macular edema (CSME), Vision Threshold DR (VTDR), stratified according to quintile categories of blood 25-hydroxyvitamin D among representative Korean adults aged 40 years or older.

	Quintile blood 25-Hydroxyvitamin D level (ng/mL)					
	13.0<(n = 425)	13.0–16.5 (n = 421)	16.5–19.6 (n = 423)	19.6–24.4 (n = 422)	24.4> (n = 422)	P for trend
**Any DR**						
Prevalence	19.3 (2.5, 14.8–24.8)	19.5 (2.9, 14.4–25.8)	21.2 (2.4, 16.8–26.2)	21.0 (2.8, 16.0–26.2)	14.8 (2.3, 10.8–20.0)	.446
Unweighted number	87	64	90	83	60	
OR						
Model 1	1.0 (reference)	1.01 (0.61–1.68)	1.12 (0.73–1.73)	1.08 (0.67–1.74)	0.70 (0.42–1.16)	.325
Model 2	1.0 (reference)	1.02 (0.60–1.73)	0.96 (0.58–1.59)	0.95 (0.58–1.69)	0.66 (0.38–1.13)	.189
**PDR**						
**Prevalence**	3.8 (1.3, 1.9–7.5)	2.5 (0.9, 1.2–4.9)	3.8 (1.0, 2.2–6.4)	2.1 (0.6, 1.1–3.8)	1.9 (0.7, 0.9–3.8)	.404
**Unweighted number**	18	10	17	15	12	
OR						
Model 1	1.0 (reference)	0.66 (0.23–1.85)	1.02 (0.39–2.67)	0.54 (0.19–1.55)	0.48 (0.17–1.29)	.204
Model 2	1.0 (reference)	0.63 (0.18–2.13)	0.92 (0.29–2.92)	0.48 (0.14–1.60)	0.52 (0.16–1.74)	.280
**CSME**						
Prevalence	1.8 (0.7, 0.8–3.7)	0.2 (0.2, 0.0–1.7)	2.0 (0.8, 0.8–4.5)	1.4 (0.6, 0.6–3.3)	1.6 (0.7, 0.7–3.7)	.312
Unweighted number	8	1	8	6	8	
OR						
Model 1	1.0 (reference)	0.13 (0.01–1.09)	1.12 (0.36–3.52)	0.81 (0.25–2.58)	0.94 (0.32–2.70)	.638
Model 2	1.0 (reference)	0.13 (0.01–1.15)	1.04 (0.32–3.41)	0.89 (0.25–3.16)	1.22 (0.40–3.72)	.344
**VTDR**						
Prevalence	6.0 (1.6, 3.6–10.0)	4.2 (1.1, 2.5–7.0)	5.3 (1.2, 3.4–8.2)	5.4 (1.6, 3.0–9.5)	3.7 (1.0, 2.1–6.3)	.697
Unweighted number	26	18	26	25	19	
OR						
Model 1	1.0 (reference)	0.68 (0.31–1.51)	0.89 (0.42–1.90)	0.90 (0.38–2.14)	0.60 (0.27–1.33)	.460
Model 2	1.0 (reference)	0.64 (0.25–1.64)	0.69 (0.28–1.27)	0.85 (0.31–2.37)	0.64 (0.25–1.59)	.570

Prevalence was expressed as weighted estimates [%] (standard errors [%], 95% confidence intervals).

Model 1: adjusted for sex and age. Model 2: adjusted for sex, age, diabetes duration, hemoglobin A1c, and hypertension. * p<0.05.

The DR prevalence rates among men and women according to the blood 25-hydroxyvitamin D quintile are shown in Table 5. In men, the prevalence of any DR significantly decreased from 20.2% to 9.5% as the blood 25-hydroxyvitamin D level increased (*P* for trend  = 0.044). In addition, the prevalence of proliferative DR correlated inversely with increasing blood 25-hydroxyvitamin D levels (*P* for trend  = 0.199). In women, however, no significant relationship was found between the DR prevalence and the 25-hydroxyvitamin D level.

**Table 5:** Gender difference of prevalence and adjusted odds ratio of any diabetic retinopathy (DR), Proliferative DR (PDR), Clinically significant macular edema (CSME), Vision Threshold DR (VTDR), stratified according to quintile categories of blood 25-hydroxyvitamin D among representative Korean adults aged 40 years or older.

	Quintile blood 25-Hydroxyvitamin D level (ng/mL)					
	Quintile 1	Quintile 2	Quintile 3	Quintile 4	Quintile 5	P for trend
**Men**						
**Range**	**14.1<(n = 212)**	**14.1–17.4 (n = 214)**	**17.4–20.5 (n = 213)**	**20.5–25.1 (n = 212)**	**25.1> (n = 212)**	
**Any DR**						
Prevalence	20.2 (3.7, 13.8–28.5)	24.5 (4.4, 17.0–34.0)	20.2 (3.9, 13.6–28.9)	19.2 (3.3, 13.4–26.6)	9.5 (2.2, 6.0–14.9)	.044*
Unweighted number	45	43	37	45	25	
OR						
Model 1	1.0 (reference)	1.28 (0.67–2.45)	0.97 (0.52–1.82)	0.91 (0.49–1.69)	0.40 (0.20–0.79)*	.013*
Model 2	1.0 (reference)	1.29 (0.62–2.66)	0.75 (0.38–1.50)	0.67 (0.33–1.39)	0.37 (0.18–0.76)*	.004*
**PDR**						
Prevalence	4.4 (2.1, 1.7–11.1)	2.9 (1.5, 1.1–7.7)	2.9 (1.4, 1.1–7.3)	1.2 (0.7, 0.4–3.4)	0.8 (0.5, 0.2–3.1)	.199
Unweighted number	10	5	7	4	2	
OR						
Model 1	1.0 (reference)	0.63 (0.15–2.66)	0.61 (0.15–2.49)	0.25 (0.06–1.04)	0.15 (0.02–0.88)*	.021*
Model 2	1.0 (reference)	0.41 (0.08–2.02)	0.50 (0.10–2.33)	0.21 (0.04–0.95)*	0.15 (0.03–0.83)*	.043*
**CSME**						
Prevalence	1.2 (0.7, 0.4–3.6)	0.6 (0.6, 0.1–4.1)	1.9 (1.2, 0.5–6.5)	1.7 (1.0, 0.6–5.0)	1.4 (0.9, 0.4–4.9)	.817
Unweighted number	4	1	3	4	3	
OR						
Model 1	1.0 (reference)	0.50 (0.05–4.77)	1.56 (0.27–8.93)	1.44 (0.29–7.15)	1.19 (0.21–6.49)	.516
Model 2	1.0 (reference)	0.84 (0.08–8.57)	2.04 (0.30–13.48)	2.55 (0.47–13.76)	3.19 (0.56–17.97)	.084
**VTDR**						
Prevalence	6.9 (2.4, 3.4–13.5)	3.2 (1.5, 1.2–7.8)	7.2 (2.6, 3.5–14.3)	2.9 (1.1, 1.3–6.0)	2.2 (1.1, 0.9–5.5)	.118
Unweighted number	15	8	12	7	5	
OR						
Model 1	1.0 (reference)	0.43 (0.13–1.46)	1.04 (0.35–3.03)	0.39 (0.13–1.15)	0.29 (0.08–0.99)*	.073
Model 2	1.0 (reference)	0.28 (0.07–1.06)	0.76 (0.22–2.52)	0.30 (0.08–1.05)	0.30 (0.08–1.08)	.101
**Women**						
**Range**	12.1<(n = 210)	12.1–15.5 (n = 212)	15.5–18.6 (n = 208)	18.6–23.5 (n = 211)	23.5> (n = 209)	
**Any DR**						
Prevalence	18.0 (3.3, 12.4–25.5)	16.4 (3.4, 10.8–24.2)	22.2 (3.8, 15.7–30.5)	19.3 (4.0, 12.7–28.4)	21.8 (4.2, 14.7–31.1)	.772
Unweighted number	39	31	46	33	40	
OR						
Model 1	1.0 (reference)	0.90 (0.45–1.77)	1.29 (0.69–2.42)	1.08 (0.55–2.11)	1.21 (0.62–2.37)	.443
Model 2	1.0 (reference)	1.01 (0.47–2.19)	1.43 (0.70–2.89)	1.17 (0.56–2.44)	1.58 (0.78–320)	.177
**PDR**						
Prevalence	2.8 (1.1, 1.3–6.0)	2.5 (1.3, 0.9–6.7)	4.9 (1.6, 2.5–9.1)	2.3 (1.1, 0.9–5.6)	3.8 (1.4, 1.9–7.6)	.603
Unweighted number	8	4	12	5	12	
OR						
Model 1	1.0 (reference)	0.88 (0.24–3.24)	1.77 (0.61–5.11)	0.80 (0.23–2.77)	1.38 (0.46–4.14)	.625
Model 2	1.0 (reference)	0.82 (0.18–3.59)	2.00 (0.62–6.42)	0.78 (0.22–2.70)	1.99 (0.68–5.83)	.279
**CSME**						
Prevalence	2.6 (1.3, 1.0–6.8)	0.5 (0.5, 0.1–3.3)	1.6 (1.2, 0.4–6.5)	0.6 (0.6, 0.1–3.7)	2.3 (1.2, 0.8–6.4)	.335
Unweighted number	4	1	4	1	6	
OR						
Model 1	1.0 (reference)	0.17 (0.01–1.61)	0.62 (0.10–3.57)	0.21 (0.02–1.85)	0.96 (0.22–4.13)	.930
Model 2	1.0 (reference)	0.18 (0.20–1.76)	0.39 (0.07–2.12)	0.16 (0.01–1.36)	0.91 (0.21–3.95)	.786
**VTDR**						
Prevalence	4.8 (1.6, 2.5–9.1)	5.2 (1.8, 2.6–10.1)	6.9 (2.0, 3.9–12.0)	3.4 (1.3, 1.6–7.2)	6.8 (1.9, 3.9–11.6)	.557
Unweighted number	11	10	17	7	17	
OR						
Model 1	1.0 (reference)	1.08 (0.40–2.92)	1.48 (0.58–3.73)	0.70 (0.24–2.02)	1.46 (0.60–3.50)	.667
Model 2	1.0 (reference)	1.09 (0.36–3.34)	1.47 (0.54–3.98)	0.55 (0.17–1.83)	1.97 (0.79–4.90)	.421

Prevalence was expressed as weighted estimates [%] (standard errors [%], 95% confidence intervals).

Model 1: adjusted for sex and age. Model 2: adjusted for age, diabetes duration, hemoglobin A1c, and hypertension. * p<0.05.

Table 5 also shows the adjusted OR for DR in both men and women. In men, the adjusted OR for any DR and proliferative DR after adjusting for potential confounders were 0.37 (95% CI, 0.18–0.76; *P* for trend  = 0.004) and 0.15 (95% CI, 0.03–0.83; *P* for trend  = 0.043), respectively, among participants in the highest 25-hydroxyvitamin D quintile relative to those in the lowest quintile. In women, however, we found no significant association between the blood 25-hydroxyvitamin D level and the DR prevalence. Table 6 shows the prevalence of DR in subjects with or without vitamin D deficiency (25-hydroxyvitamin D <20 ng/mL). Prevalence of proliferative DR was significantly higher in those with vitamin D deficiency than in those without vitamin D deficiency (*P* = 0.002).

**Table 6:** Prevalence of any diabetic retinopathy (DR), Proliferative DR (PDR), Clinically significant macular edema (CSME), and Vision Threshold DR (VTDR) in subjects with or without vitamin D deficiency (25-hydroxyvitamin D<20 ng/mL).

Prevalence (%)	Vitamin D deficiency	No Vitamin D deficiency	*p*
**Both**			
** Any DR**	19.7 (1.4)	18.0 (2.0)	.494
** PDR**	3.3 (0.6)	1.9 (0.5)	.073
** CSME**	1.3 (0.4)	1.5 (0.5)	.698
** VTDR**	5.1 (0.7)	4.5 (1.0)	.638
**Men**			
** Any DR**	21.0 (2.4)	16.1 (2.3)	.128
** PDR**	3.6 (1.0)	0.9 (0.4)	.002*
** CSME**	1.3 (0.5)	1.4 (0.6)	.855
** VTDR**	5.0 (1.2)	3.7 (1.2)	.441
**Women**			
** Any DR**	18.3 (1.8)	21.2 (3.2)	.419
** PDR**	3.0 (0.7)	3.7 (1.0)	.586
** CSME**	1.4 (0.5)	1.7 (0.8)	.675
** VTDR**	5.1 (0.9)	5.8 (1.3)	.644

Data are expressed as weighted means or weighted frequency (%) with standard errors. * p<0.05.

## Discussion

Our study demonstrated that the risks of any DR and proliferative DR decreased in those with high blood 25-hydroxyvitamin D levels relative to those with the lowest blood 25-hydroxyvitamin D levels. Additionally, this association between blood 25-hydroxyvitamin D levels and DR was only observed in men.

We found that in men, the odds of any DR were 63% lower among subjects in the highest blood 25-hydroxyvitamin D quintile relative to those in the lowest quintile after adjusting for age, diabetes duration, hemoglobin A1c levels, and hypertension. Our finding contradicts that of the only previous epidemiologic study, which examined the relationship between blood 25-hydroxyvitamin D levels and DR. The previous study showed no significant association between the 2 variables, despite finding an association between DR severity and the prevalence of vitamin D deficiency. One possible reason for this discrepancy is the difference in average blood 25-hydroxyvitamin D levels between the 2 studies. The median (interquartile range) blood 25-hydroxyvitamin D concentration in the present study was 17.7 (13.8–22.5) ng/mL, which was lower than that reported in the U.S. NHANES (25.8 [17.9–31.1] ng/mL). However, it is unknown that low vitamin D levels has more strong association with DR. Ethnic factors might explain this discrepancy. The serum vitamin D levels are reportedly lower in Asian populations than in Caucasian populations [34]. In addition, the prevalence of vitamin D deficiency might have increased during the 20-year interval between the 2 studies. Globally, vitamin D deficiency has reportedly increased during the past few decades [35]. Industrialization has reduced the exposure of skin to sunlight, by which means approximately 90% of vitamin D is generated [7]. Another possible cause of this discrepancy is the that there could be methodological difference in vitamin D measurement, because the U.S. NHANES III was evaluated nearly 20 years ago.

Moreover, our study found that the risk of proliferative DR decreased by 85% in those with the highest blood 25-hydroxyvitamin D levels relative to those with the lowest levels after adjusting for potential confounders in men. One possible biological explanation for this finding is that vitamin D inhibits angiogenesis, which is among the most critical characteristics of proliferative DR. Vitamin D inhibits angiogenesis by reducing vascular endothelial growth factor expression, reducing endothelial cell proliferation, and increasing platelet-derived growth factor expression [6], [8], [16]. Moreover, vitamin D also inhibits matrix-metalloproteinase 9, which plays a role in DR development [36], [37]. Our findings warranted need for interventional study with dietary vitamin D supplementation in diabetic patients to evaluate any possible effect of vitamin D on DR development and progression.

The present study found a sex-related difference in the association between blood 25-hydroxyvitamin D levels and DR. Although men with high blood 25-hydroxyvitamin D levels had 63% and 85% lower risks of any DR and proliferative DR, respectively, relative to those with the lowest levels after adjusting for potential confounders, similar associations were not observed in women. Moreover, a sex-related effect modification was observed. The directions of the OR for any DR and proliferative DR in women (OR = 1.58 and 1.99, respectively) were opposite those in men (OR = 0.37 and 0.15, respectively), which would explain the lack of an overall association (Table 4). Our findings suggest that hypovitaminosis D might be a significant risk factor for any DR and proliferative DR in men but not in women. The lack of an inverse correlation between the vitamin D status and DR in women is interesting, particularly after accounting for the lower levels of vitamin D levels in women relative to those in men. A study reported gender-specific association of vitamin D receptor polymorphism which has significant effect on diabetes [38]. It is unclear why 25-hydroxyvitamin D is associated with any DR and proliferative DR in men but not in women. The variable threshold effect between the sexes might provide an explanation for the lack of an association. It is also possible that women exhibit a decreased sensitivity to vitamin D. However, this is purely speculative. Further studies are needed to identify the factors responsible for this difference and especially to elucidate the exact sex-related biologic mechanisms. Given the data currently in this study, any future clinical trials need to focus on men, and further observation data exploration would be useful to help determine which findings are real between those associated with men or those with women.

The major strengths of the present study are the relatively large number of participants with diabetes (n = 2,553) and the study design that incorporated systemically stratified, multistage, clustered random sampling methods. Another strength is the rigorous quality control regarding ophthalmic fundus examinations and blood 25-hydroxyvitamin D measurements in KNHANES. Finally, DR was evaluated using 7 standard photographs obtained under pharmacological dilatation. Our study also has several limitations. First, we could not adjust for seasonal variations in the 25-hydroxyvitamin D levels because KNHANES did not include information about the examination dates. Although a recent study showed that Asian populations displayed no significant seasonal vitamin D status variations [34], effect of seasonal variation in vitamin D may affect in absence of a consistent trend between the quintiles. Second limitation is that our study featured a cross-sectional design that introduced difficulties when inferring causality. Third, multiple comparisons were done, because gender was found to be effect modifier. Thus, the results stratified by gender were presented separately. We declare that the findings of an association between retinopathy and vitamin D in men only is at best hypothesis-generating. Finally, as dietary intervention of vitamin D was not considered in the present study, 25-hydroxyvitamin D2 and 25-hydroxyvitamin D3 may not have equivalent physiological actions, and natural (mainly via sunlight) and dietary sources differ in being predominantly 25-hydroxyvitamin D2 or 25-hydroxyvitamin D3.

In conclusion, the present study provides the population-based epidemiologic finding of an inverse relationship between the absolute blood 25-hydroxyvitamin D levels and DR in a representative Korean population. The blood 25-hydroxyvitamin D level correlated inversely with the prevalence of any DR and proliferative DR in men but not in women. It could be speculated that 25-hydroxyvitamin D may exert a inhibitory effect on both DR development and progression via its anti-angiogenic and anti-inflammatory actions. Considering that the prevalence of vitamin D deficiency has increased worldwide in the past few decades, further studies including longitudinal and interventional studies are warranted.

## References

[1] Kobrin Klein BE (2007) Overview of epidemiologic studies of diabetic retinopathy. Ophthalmic epidemiology 14:179–183. 17896294 10.1080/09286580701396720

[2] Yau JW, Rogers SL, Kawasaki R, Lamoureux EL, Kowalski JW, et al (2012) Global prevalence and major risk factors of diabetic retinopathy. Diabetes Care 35:556–564. 22301125 10.2337/dc11-1909 PMC3322721

[3] Jee D, Lee WK, Kang S (2013) Prevalence and risk factors for diabetic retinopathy: the Korea National Health and Nutrition Examination Survey 2008–2011. Invest Ophthalmol Vis Sci 54:6827–6833. 24065813 10.1167/iovs.13-12654

[4] Van Leiden HA, Dekker JM, Moll AC, Nijpels G, Heine RJ, et al (2002) Blood Pressure, Lipids, and Obesity Are Associated With Retinopathy The Hoorn Study. Diabetes care 25:1320–1325. 12145228 10.2337/diacare.25.8.1320

[5] Klein BE, Klein R, McBride PE, Cruickshanks KJ, Palta M, et al (2004) Cardiovascular disease, mortality, and retinal microvascular characteristics in type 1 diabetes: Wisconsin Epidemiologic Study of Diabetic Retinopathy. Archives of internal medicine 164:1917. 15451768 10.1001/archinte.164.17.1917

[6] Albert DM, Scheef EA, Wang S, Mehraein F, Darjatmoko SR, et al (2007) Calcitriol is a potent inhibitor of retinal neovascularization. Invest Ophthalmol Vis Sci 48:2327–2334. 17460298 10.1167/iovs.06-1210

[7] Palomer X, González-Clemente J, Blanco-Vaca F, Mauricio D (2008) Role of vitamin D in the pathogenesis of type 2 diabetes mellitus. Diabetes, Obesity and Metabolism 10:185–197. 10.1111/j.1463-1326.2007.00710.x 18269634

[8] Chung I, Han G, Seshadri M, Gillard BM, Yu W-d, et al (2009) Role of vitamin D receptor in the antiproliferative effects of calcitriol in tumor-derived endothelial cells and tumor angiogenesis in vivo. Cancer research 69:967–975. 19141646 10.1158/0008-5472.CAN-08-2307 PMC2752059

[9] Pittas AG, Harris SS, Stark PC, Dawson-Hughes B (2007) The effects of calcium and vitamin D supplementation on blood glucose and markers of inflammation in nondiabetic adults. Diabetes care 30:980–986. 17277040 10.2337/dc06-1994

[10] Mangge H, Weghuber D, Prassl R, Haara A, Schnedl W, et al (2013) The Role of Vitamin D in Atherosclerosis Inflammation Revisited: More a Bystander than a Player? Curr Vasc Pharmacol 10.2174/1570161111666131209125454 24329737

[11] Firrincieli D, Braescu T, Housset C, Chignard N (2014) Illuminating liver fibrosis with vitamin D. Clin Res Hepatol Gastroenterol 38:5–8. 24238723 10.1016/j.clinre.2013.10.004

[12] Yilmaz SS, Hizli D, Yilmaz E, Eryilmaz OG, Hizli F, et al (2013) Effect of vitamin D on postoperative adhesion formation in a rat uterine horn adhesion model. J Reprod Med 58:511–516. 24568046

[13] Wang Q, He Y, Shen Y, Zhang Q, Chen D, et al (2014) Vitamin D inhibits COX-2 expression and inflammatory response by targeting thioesterase superfamily member 4. J Biol Chem 10.1074/jbc.M113.517581 PMC4002078 24619416

[14] Chen J, Bruce D, Cantorna MT (2014) Vitamin D receptor expression controls proliferation of naive CD8+ T cells and development of CD8 mediated gastrointestinal inflammation. BMC Immunol 15:6. 24502291 10.1186/1471-2172-15-6 PMC3923390

[15] Havakuk O, Entin-Meer M, Ben-Shoshan J, Goryainov P, Maysel-Auslender S, et al (2013) Effect of vitamin D analogues on acute cardiorenal syndrome: a laboratory rat model. Isr Med Assoc J 15:693–697. 24511650

[16] Toriola AT, Nguyen N, Scheitler-Ring K, Colditz GA (2014) Circulating 25-hydroxyvitamin D (25-OHD) levels and prognosis among cancer patients: a systematic review. Cancer Epidemiol Biomarkers Prev 10.1158/1055-9965.EPI-14-0053 24692501

[17] Quraishi MK, Badsha H (2013) Rheumatoid arthritis disease activity and vitamin D deficiency in an Asian resident population. Int J Rheum Dis 10.1111/1756-185X.12209 24261616

[18] Autier P, Boniol M, Pizot C, Mullie P (2014) Vitamin D status and ill health: a systematic review. Lancet Diabetes Endocrinol 2:76–89. 24622671 10.1016/S2213-8587(13)70165-7

[19] Lavie CJ, Dinicolantonio JJ, Milani RV, O'Keefe JH (2013) Vitamin D and cardiovascular health. Circulation 128:2404–2406. 24276875 10.1161/CIRCULATIONAHA.113.002902

[20] Taverna MJ, Selam J-L, Slama G (2005) Association between a protein polymorphism in the start codon of the vitamin D receptor gene and severe diabetic retinopathy in C-peptide-negative type 1 diabetes. Journal of Clinical Endocrinology & Metabolism 90:4803–4808. 15899948 10.1210/jc.2004-2407

[21] Joussen AM, Poulaki V, Le ML, Koizumi K, Esser C, et al (2004) A central role for inflammation in the pathogenesis of diabetic retinopathy. The FASEB journal 18:1450–1452. 15231732 10.1096/fj.03-1476fje

[22] Simo R, Carrasco E, Garcia-Ramirez M, Hernandez C (2006) Angiogenic and antiangiogenic factors in proliferative diabetic retinopathy. Current diabetes reviews 2:71–98. 18220619 10.2174/157339906775473671

[23] Payne JF, Ray R, Watson DG, Delille C, Rimler E, et al (2012) Vitamin D insufficiency in diabetic retinopathy. Endocrine Practice 18:185–193. 21940279 10.4158/EP11147.OR PMC4706181

[24] Aksoy H, Akçay F, Kurtul N, Baykal O, Avci B (2000) Serum 1, 25 dihydroxy vitamin D (1, 25 (OH)<sub> 2</sub> D<sub> 3</sub>), 25 hydroxy vitamin D (25 (OH) D) and parathormone levels in diabetic retinopathy. Clinical biochemistry 33:47–51. 10693986 10.1016/s0009-9120(99)00085-5

[25] Kim Y, Park S, Kim NS, Lee BK (2013) Inappropriate survey design analysis of the Korean National Health and Nutrition Examination Survey may produce biased results. J Prev Med Public Health 46:96–104. 23573374 10.3961/jpmph.2013.46.2.96 PMC3615385

[26] Park HA (2013) The Korea national health and nutrition examination survey as a primary data source. Korean J Fam Med 34:79. 23560205 10.4082/kjfm.2013.34.2.79 PMC3611106

[27] American Diabetes Association (2002) Report of the expert committee on the diagnosis and classification of diabetes mellitus. Diabetes Care 23 Suppl 1 S4–19. 12017675

[28] Wong TY, Klein R, Islam FA, Cotch MF, Folsom AR, et al (2006) Diabetic retinopathy in a multi-ethnic cohort in the United States. American journal of ophthalmology 141:446. 16490489 10.1016/j.ajo.2005.08.063 PMC2246042

[29] Group DRS (1981) Diabetic retinopathy study. Report Number 7. A modification of the Airlie House classification of diabetic retinopathy. Investigative ophthalmology & visual science 21:6. 7195893

[30] Eum K-D, Lee M-S, Paek D (2008) Cadmium in blood and hypertension. Science of the Total Environment 407:147–153. 18845316 10.1016/j.scitotenv.2008.08.037

[31] Lee M-S, Park SK, Hu H, Lee S (2011) Cadmium exposure and cardiovascular disease in the 2005 Korea National Health and Nutrition Examination Survey. Environmental research 111:171–176. 21055738 10.1016/j.envres.2010.10.006 PMC3683977

[32] Sempos CT, Vesper HW, Phinney KW, Thienpont LM, Coates PM (2012) Vitamin D status as an international issue: national surveys and the problem of standardization. Scand J Clin Lab Invest Suppl 243:32–40. 10.3109/00365513.2012.681935 22536760

[33] Kim K (2012) Blood cadmium concentration and lipid profile in Korean adults. Environmental research 112:225–229. 22208951 10.1016/j.envres.2011.12.008

[34] Smith M (2010) Seasonal, ethnic and gender variations in serum vitamin D3 levels in the local population of Peterborough. Bioscience Horizons 3:124–131.

[35] Personne V, Partouche H, Souberbielle JC (2013) [Vitamin D insufficiency and deficiency: epidemiology, measurement, prevention and treatment]. Presse Med 42:1334–1342. 24051167 10.1016/j.lpm.2013.06.013

[36] Bahar-Shany K, Ravid A, Koren R (2010) Upregulation of MMP-9 production by TNFα in keratinocytes and its attenuation by vitamin D. Journal of cellular physiology. 222:729–737. 10.1002/jcp.22004 20020446

[37] Timms P, Mannan N, Hitman G, Noonan K, Mills P, et al (2002) Circulating MMP9, vitamin D and variation in the TIMP-1 response with VDR genotype: mechanisms for inflammatory damage in chronic disorders? Qjm 95:787–796. 12454321 10.1093/qjmed/95.12.787

[38] Gyorffy B, Vásárhelyi B, Krikovszky D, Madácsy L, Tordai A, et al (2002) Gender-specific association of vitamin D receptor polymorphism combinations with type 1 diabetes mellitus. European journal of endocrinology 147:803–808. 12457456 10.1530/eje.0.1470803

